# Brain‐Behavior Associations During Interactions Between Caregivers and Infants

**DOI:** 10.1111/infa.70044

**Published:** 2025-09-03

**Authors:** Aimee Theyer, Sobanawartiny Wijeakumar

**Affiliations:** ^1^ School of Psychology University of Nottingham Nottingham UK

**Keywords:** caregiver‐infant interactions, fNIRS, temporal cortex, visual short‐term memory

## Abstract

Previous research has shown that infants' abilities to sustain attention are influenced by caregivers' attentional behaviors. Here, we inquired whether brain function in infants was linked to brain function in caregivers during attention periods in dyadic interactions, and whether this brain function was associated with visual short‐term memory in infants. Caregivers (*n* = 90, mean age = 33.5 years) and infants (*n* = 91, mean age = 251.3 days) were recorded for 5‐ to 7‐min as they naturalistically played with toys. During these interactions, brain function was recorded using functional near‐infrared spectroscopy (fNIRS). To assess visual short‐term memory, infants were presented with a preferential looking task. Video recordings were coded for periods of joint attention between caregivers and infants, and periods of continued attention in infants. fNIRS data was processed to extract significant clusters of activation. Our findings revealed that temporo‐parietal engagement in both caregivers and infants. Specifically, left superior temporal gyrus activation in caregivers during joint attention was linked to duration of joint attention, duration of continued attention, and visual short‐term memory in infants. Our findings highlight cortical mechanisms engaged in caregivers and infants during dyadic interactions, and importantly, how these mechanisms are linked to visual short‐term memory.

## 1 Introduction

2As early as the first few months of life, infants learn to direct, engage, and re‐orient their attention (Aslin and Salapatek 1975; Johnson et al. 1991; Richards 2001) and exhibit novelty preferences, habituation to repetitive stimulation, and distraction (Ruff and Capozzoli 2003; Colombo 2001). There is also a wealth of evidence demonstrating the role of caregiver input on infant object engagement (Michel et al. 2024; Wass, Clackson, et al. 2018; Hoehl et al. 2012) and attention (D’Entremont 2000; Hood et al. 1998; Farroni et al. 2000) in the first year of life. For example, joint visual attention between caregivers and infants when interacting with an object is related to the duration of visual attention that infants subsequently paid to the same object (Yu and Smith 2016). In addition to mutual gaze, caregivers and infants show attention‐sharing during object manipulation (Yu and Smith 2013; Deák et al. 2014) and related verbalizations (Schroer and Yu 2022). This type of “multimodal” input, referring to the combination of caregiver touch, gesturing and verbalizations is associated with longer play bouts in infants (Schatz et al. 2022). Collectively, these findings indicate that caregiver engagement combining gaze, object‐associated manipulation, and verbalizations impact infant engagement during interactions.

Whilst there is a wealth of research investigating infant and caregiver engagement during social interactions, changes in underlying brain function in either partner is lesser explored. Most previous research has focussed on adult studies (Materna et al. 2008; Morris et al. 2005; Bristow et al. 2007; Williams et al. 2005; Schilbach et al. 2006; Redcay et al. 2012). Existing literature posits that joint attention occurs as an outcome of posterior and anterior attention systems in the brain (Mundy and Newell 2007). The posterior attention system, composed of parietal and superior temporal regions, somatosensory association cortex, angular gyrus, superior temporal gyrus, and auditory cortex, is thought to prioritize orientation toward relevant stimuli, alongside imitation and perception of others. The anterior attention system involves the frontal eye fields, dorsolateral prefrontal cortex, orbitofrontal area, pars orbitalis and anterior cingulate cortex and is thought to be involved in goal‐directed attentional allocation. Together, these systems are purported to support joint attention (Williams et al. 2005; Redcay et al. 2012; Mundy and Newell 2007; Mundy et al. 2000; Mundy 2003; Posner and Rothbart 2007). There is limited evidence that infants activate similar networks during joint engagement. For example, a study examining brain function in 5‐month‐old infants during interactions with a virtual social partner reported that infants recruited the prefrontal cortex when their partner followed their own gaze (Grossmann et al. 2013). In another study where a live experimenter played the role of the social partner with 7‐ to 12‐month‐old infants, the authors reported activation in the bilateral dorsal prefrontal cortex in the infants when initiating joint attention, and right‐lateralized prefrontal activation when initiating and responding to joint attention (Naoi et al. 2022). In a similar vein in toddlers, initiating joint attention bids has been linked to left frontal and central cortical activity, and responding to joint attention bids has been linked to left parietal activation and right parietal deactivation (Mundy et al. 2000). Whilst these studies make important contributions to the understanding of brain function in infants during joint attention, they have used structured tasks, and/or paired infants with a virtual partner/experimenter instead of caregivers, thereby, reducing ecological validity necessary for contextualizing this developmental period.

Existing work investigating brain function in both caregivers and infants during interactions mostly focuses on examining interbrain synchrony (Leong et al. 2017; Papoutselou et al. 2024; Marriott Haresign et al. 2023; Nguyen et al. 2020). Only a handful of studies have examined cortical underpinnings of attention—and/or cognition‐related processes in both caregivers and infants during these interactions. For example, a study using electroencephalograms (EEG) reported that theta power in infants predicted greater visual attention in solo play compared to joint play (Wass, Noreika, et al. 2018). The authors also reported that during joint play, infants were generally more attentive, and caregiver theta power closely tracked changes in infant attention. Further, greater neural responsivity in caregivers was also associated with greater infant sustained attention periods. Another study examined the impact of caregiver ostensive signals on infant neural activity and object encoding (Michel et al. 2024). Here, the use of ostensive signals increased infants' attention to both the caregiver and the object. Notably, greater theta activity during these interactions also predicted better object encoding, suggesting that social engagement with caregivers enhances infants' ability to process and learn about objects. More research is necessary in this direction to understand how cortical mechanisms are differentially engaged during types of attention periods, and how these mechanisms might be related in caregivers and their infants.

Extent of caregiver‐infant engagement during these attention periods is linked to aspects of infant and child cognitive development. Previous work has shown that both joint attention in caregivers and infants and sustained attention in infants predict vocabulary size in toddlerhood, with sustained attention within the context of joint attention yielding stronger predictions, compared to joint attention by itself (Yu et al. 2019). Joint attention in infancy has also been linked to gestural abilities (Carpenter et al. 1998), language development (Carpenter et al. 1998; Morales et al. 2000), and individual differences in IQ, self‐regulation, and social abilities (Mundy, Block, et al. 2007). However, to our knowledge, no studies have explored the link between brain‐behavior associations during these attention periods, and visual cognition in infancy using objective experimental methods.

To address afore‐mentioned issues, the current study aims to examine brain function in infants and caregivers during critical attention periods in dyadic interactions, and whether extent of engagement and brain function during these periods are associated with visual short‐term memory in infants. To this end, we collected video recordings and brain function using functional near‐infrared spectroscopy (fNIRS) from caregivers and 6‐to‐10‐month‐old infants while they naturalistically engaged each other with toys. We focussed on this age group because previous work has shown that gaze‐following and joint attention abilities emerge around this developmental period (Scaife and Bruner 1975; Striano et al. 2006; Striano and Stahl 2005). Further, most research on dyadic interactions has focussed on later infancy and toddlerhood (Yu and Smith 2013, 2016; Schroer and Yu 2022; Schatz et al. 2022; Herzberg et al. 2022; Tamis‐LeMonda and Bornstein 1991; Suarez‐Rivera et al. 2022; Rachwani et al. 2020). During these dyadic interactions, we focussed on periods of joint attention between caregivers and infants (we refer to this as “joint attention” from here on) and periods of infant attention subsequently following joint attention (we refer to this as “continued attention” from here on). Lastly, to measure visual short‐term memory in infants, we presented infants with a preferential looking task.

The current study had five research objectives. The first objective was to examine whether duration of joint attention was associated with duration of continued attention in infants. Based on previous findings reporting a positive link between proportion of joint attention and sustained attention in caregivers and toddlers (Yu and Smith 2016), we expected to observe a similar positive link between joint attention and continued attention. The second objective was to identify key brain regions engaged by caregivers and infants during these attention periods. Based on previous evidence of brain regions involved in joint attention (Mundy and Newell 2007), we expected anterior‐posterior, more specifically, fronto‐parietal engagement during attention episodes. The third objective was to examine whether there was an association between brain function in caregivers and brain function in infants during joint attention and, how this brain function was linked to duration of joint attention. The fourth objective was to examine whether duration of continued attention in infants was associated with brain function in infants during continued attention, brain function in infants during joint attention, and brain function in caregivers during joint attention. To our knowledge, no study has examined the link between caregiver and child brain function during these attention periods, and links to duration of these periods, and thus, we conducted exploratory analyses here. The last objective examined whether key behavior and brain function measures emerging from meeting afore‐mentioned objectives were associated with visual short‐term memory in infants. In line with evidence generally linking both joint attention and sustained attention to cognitive outcomes (Yu et al. 2019), we expected that both behavior and brain measures during joint attention and continued attention would be linked to infant visual short‐term memory.

## 2 Methods

### 2.1 Participants

This study is part of a larger project. To determine sample size necessary for this study, calculations were performed with an effect size of 0.15, alpha = 0.05 and power = 0.80 (*F*‐tests for linear multiple regression using the A priori method for three predictors). These calculations revealed that a sample size of 77 was required. To meet this sample size, families were recruited via advertisement on social media platforms and by distributing study information among schools, nurseries, shops and baby and toddler groups. One hundred eleven families with 6‐to‐10‐month‐old infants expressed interest and were provided a detailed explanation of the study. Next, an eligibility check was conducted. Families were excluded if (1) they did not have normal or corrected‐to‐normal vision, (2) they had any neurological conditions or had experienced major head trauma, (3) English was not the primary language spoken at home, (4) the caregiver had illicit drug or alcohol usage during the pregnancy, (5) the pregnancy term was not between 37 and 42 weeks, or (6) there were complications in the birth. 17 families dropped out of the study and 4 families were not included as the infant was not in the correct age range, resulting in 90 families who visited the lab (90 caregivers: 89 females and 1 non‐binary, mean age: 33.50 ± 4.45 years, age range: 22–46 years; 91 infants: 46 females, mean age: 251.3 days ± 34.98, age range: 185–318 days, including one pair of twins). Demographic and socioeconomic status information is provided in Table 1.

**TABLE 1:** Socioeconomic and demographic information was collected via questionnaire after the family visited the research facility.

Measure	*N*	Mean	SD
Infant age (days)	91	251.30	34.98
Primary caregiver age	90	33.50	4.45
Primary caregiver education (years)	78	16.23	2.34
Secondary caregiver education (years)	77	13.83	4.32
Primary caregiver income	71	£27,638	£13,349
Secondary caregiver income	51	£31,504	£11,669

Primary caregiver's ethnicity	*N*	%
Arab	1	1
Asian	5	6
Mixed	4	4
White British	67	74
Other White	1	1
Did not answer	12	13

Ethics approval was provided by the Research Ethics Committee at the University of Nottingham (Ethics Approval Number: F1415). Caregivers and infants were invited to visit the Infant and Toddler Lab at the University of Nottingham in the United Kingdom. Upon arrival, the procedures were explained to the caregiver. Informed consent was obtained from the caregivers for their participation and on behalf of the participation of their infant.

### 2.2 Caregiver‐Infant Dyadic Interactions

#### 2.2.1 Data Collection

**FIGURE 1:**
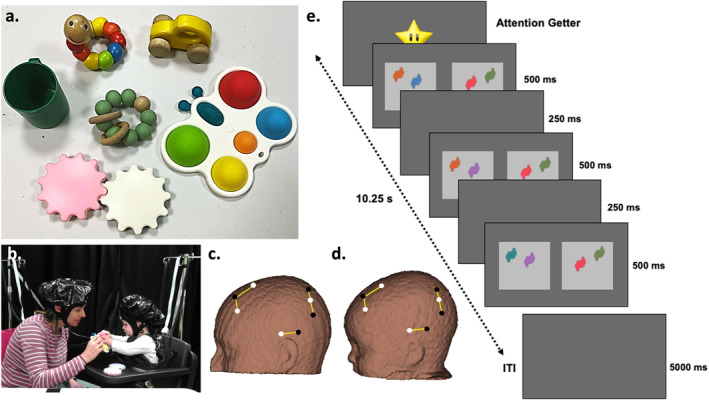
(a) Toys used in dyadic interaction and (b) example of the dyadic set‐up. fNIRS probe geometry with channels covering frontal, temporal, and parietal regions used for (c) caregivers and (d) infants during the dyadic interactions. White dots indicate sources, black dots indicate detectors, and yellow connections indicate channels. (e) Infant visual short‐term memory task at the medium load. Changing side is shown on the left.

For the dyadic interaction, caregivers sat on a chair and infants were either seated in a highchair with a tray attachment, opposite their caregivers or on their caregiver's lap, if they were fussy. Caregivers were asked to naturalistically engage their infants with a selection of 6 objects (butterfly, car, gears, teething ring, cup, and worm)—see Figure 1a,b. These toys were selected because they were appropriate for infants between the ages of 6 and 10 months and were frequently purchased by families, signifying comfort with use.

fNIRS data was also collected from both caregivers and infants during dyadic interactions using a NIRScout System (wavelengths 760 and 850 nm). The probe geometry consisted of 8 sources and 8 detectors each for caregivers and infants, creating 10 channels overlaying the frontal, temporal and parietal cortices (Figure 1c,d). Multiple cap sizes were used to accommodate for the varying head sizes (caregivers: 54, 56, and 58 cm and infants: 42, 44, 46, 48, 50, 52 cm). Source‐detector separation was scaled according to cap size used (e.g., 48 cm: 2.4 cm, 50 cm: 2.5 cm, 52 cm: 2.6 cm, 54 cm: 2.7 cm, 56 cm: 2.8 cm, 58 cm: 2.9 cm). When the caregiver was ready, the experimenters placed the appropriately sized fNIRS cap onto the caregivers' head. Following this, the experimenters then placed an appropriately sized fNIRS cap onto the infants' head and commenced the interaction. Throughout, one experimenter was responsible for presenting the task and monitoring the interaction using the video recordings and the other experimenter monitored the fNIRS signals.

Two video cameras were used to record the interaction. One video camera was placed closer to the dyad and offered a better view of the objects on the table, and another video camera was placed further away from the dyad to capture the full scene. These recordings were captured using Bandicam (https://www.bandicam.com/) and Movavi programs (https://www.movavi.com/mac/) and presented in real‐time to the experimenter so that they could monitor the interactions. In addition, when possible, caregivers wore a head‐mounted camera (Kassner et al. 2014). This video was used as a third angle to confirm details that were not evident from the other two video recordings. PsychoPy software (v2021.2.3) was used to generate a flash and an audio tone on a TV to indicate the start and end of the interaction.

The play interaction lasted for a maximum of 7 min. However, some interactions were terminated earlier if the infant became fussy (6 min 15 s for one family and 5 min 20 s for one family).

#### 2.2.2 Coding Joint Attention and Continued Attention

Caregiver and infant engagement during the play interactions were coded using DataVyu software (https://datavyu.org/). Each play interaction was divided into 5‐s epochs. Each epoch was coded into one of 9 engagement categories for each infant and caregiver separately (see Table 2 for codes and descriptions). There was no minimum duration set for a behavior to be assigned a code. If the infant/caregiver demonstrated a behavior during the 5‐s epoch, then this epoch was coded as the corresponding engagement category. Note, however, that if an infant/caregiver demonstrated more than one behavior (e.g., looking and touching), the epoch was coded as another engagement category (e.g., LT). Therefore, the code assigned reflected only behaviors that occurred during the epoch, excluding the occurrence of any of the other behaviors. For instance, an epoch with code “L” will not contain intentional touches or verbalizations/vocalizations for any duration.

**TABLE 2:** Behavior codes and their descriptions.

Code	Description
L^a^	*Look*: Caregiver/infant gazes toward the object during the epoch.
T	*Touch*: Caregiver/infant makes any intentional manual contact with the object during the epoch.
V	*Verbalization* (caregivers only): The caregiver makes any object‐specific verbalizations during the epoch ‐ naming the object, describing object properties, describing actions that could be completed with the object, or providing a narrative associated with the object (e.g., “look at the butterfly”, “can you point toward the green one?”, “these can spin around”, “we were driving earlier today”).
	*Vocalization* (infants only): The infant vocalizes or babbles whilst engaging with the object (e.g., “ah”, “ooh”). This does not include general noises such as sighing, crying, or laughing.
LT^a^	Caregiver/infant demonstrated both *look* and *touch* behaviors during the epoch.
LV	Caregiver/infant demonstrated both *look* and *verbalization/vocalization* behaviors during the epoch.
TV	Caregiver/infant demonstrated both *touch* and *verbalization/vocalization* behaviors during the epoch.
LTV^a^	Caregiver/infant demonstrated *look*, *touch*, and *verbalization/vocalization* behaviors during the epoch
X	Exclusion: Caregiver/infant does not show any look, touch, or verbalization/vocalization behaviors with any object during the interaction.
N	Exclusion: Other reasons such as experimenter interrupts during the epoch, infant is distracted by cap/wires or fussy for greater than half of the epoch.

^a^ Indicates unimodal, bimodal, and multimodal engagement periods used for defining joint and continued attention periods.

For infants, L accounted for 12.2%, LT for 80.3%, and LTV for 3.5% of the interactions, and for caregivers, L accounted for 15%, LT for 61.6%, and LTV for 20.9% of the interactions. For both infants and caregivers, all other categories (LV, T, TV, and V) each accounted for less than 2.4% of the interactions. Therefore, only L (referred to as unimodal), LT (referred to as bimodal), and LTV (referred to as multimodal) codes were considered for defining joint and continued attention periods. Two coders were trained to code this data. One coder coded all participants, and another coder coded 23% of the data. Reliability was computed using Cohen's Kappa, a statistic that looks at percent agreement across categories normed by the base rate of each category. Kappa values from 0.81 to 0.99 indicate near perfect agreement. For caregiver data, the mean Kappa value was 0.96 and for infant data, the mean Kappa value was 0.96.

**FIGURE 2:**
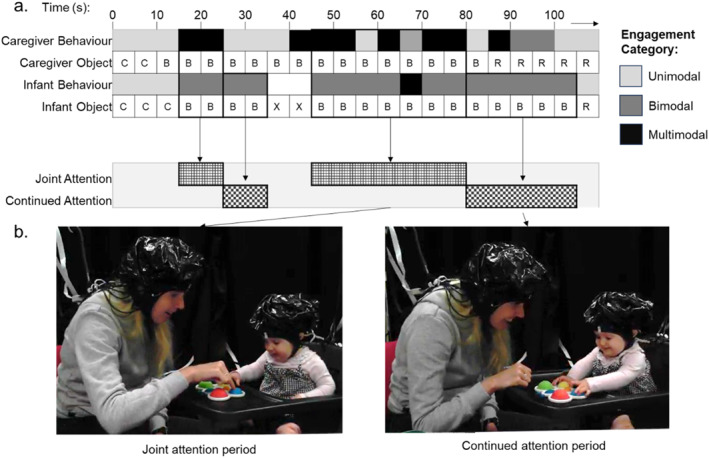
(a) Joint and continued attention periods extracted based on caregiver and infant behaviors. (b) Example of a dyad during these periods.

For the current study, a “joint attention” period was defined as at least two concurrent epochs during which caregivers were multimodally engaging (LTV) with an object that their infant was either unimodally, bimodally, or multimodally (L, LT, and/or LTV) engaging with. To code these periods, we first coded the object(s) that caregivers and infants interacted with during each epoch (butterfly: code “B”, car: code “C”, gears: code “G”, teething ring: code “R”, cup: code “S”, and worm: code “W”). If infants interacted with more than one object, this epoch was coded as a combination of codes (e.g., BC). Next, we identified periods during which caregivers showed multimodal engagement with one object for at least two concurrent epochs and infants showed unimodal, bimodal, or multimodal engagement with the same object (Figure 2a). Note that for infants, we also included epochs if they showed some engagement with another object, so long as they were still interacting with the object of interest. The joint attention period ended if the caregiver switched to a different type of engagement for more than one epoch and/or if the infant did not unimodally, bimodally, or multimodally engage with the same object anymore. We focused on multimodal engagement for the caregiver because previous work has shown that multimodal engagement is linked to infant play duration and complexity (Schatz et al. 2022; Suarez‐Rivera et al. 2022; Suarez‐Rivera et al. 2019). Further, since caregivers could jointly attend with their infants in several ways across all modalities, we wanted to define determinate, distinguishable periods. We set a criterion of at least 2 concurrent epochs (or 10 s) for specifying a joint attention period for the purpose of examining underlying brain function during this period. This criterion was drawn from experimental fNIRS studies which typically use event windows of 5–10 s to capture the hemodynamic response.

A “continued attention” period was defined as epochs following a joint attention period, during which caregiver multimodal engagement had ended and the infant continued to unimodally, bimodally, or multimodally engage with the same object they had interacted with during joint attention (Figure 2b). We also included epochs during which infants showed some engagement with another object, so long as they were still interacting with the object of interest. The continued attention period ended if the infant no longer showed unimodal, bimodal, or multimodal engagement with the object from the preceding joint attention period and/or a third object was introduced, and/or if the caregiver re‐joined the interaction with multimodal engagement such that a new joint attention period began. We decided to focus on continued attention periods following multimodal joint attention because previous work has shown that caregiver joint attention (Yu and Smith 2016; Yu et al. 2019), particularly multimodal input (Schatz et al. 2022; Suarez‐Rivera et al. 2022; Suarez‐Rivera et al. 2019) is associated with extended infant sustained attention.

We extracted duration of joint attention periods in seconds from caregivers and infants, and continued attention periods in seconds from infants. The mean period length was 18.48 s for joint attention, and 21.97 s for continued attention. Duration was summed across periods per dyad and used in further analyses. Summary statistics are reported in Supporting Information S1: Table S1.

#### 2.2.3 fNIRS Neuroimaging Data Analyses

##### 2.2.3.1 Pre‐Processing fNIRS Data

The start onsets of joint attention periods and continued attention periods were inserted into caregiver and infant fNIRS data to indicate the start of the duration of each period. Caregivers and infants contributed an average of around 3 events each for both joint attention and continued attention. All fNIRS data was pre‐processed in *EasyNIRS* using Homer2. First, raw data was pruned to remove noisy channels (dRange = 0.03–2.0; SNRthresh = 2; caregiver SDrange = 2.5–4.5; and infant SDrange = 2–3). 0.62% of the infants' and 0.48% of the caregivers' channels were pruned. Then, raw intensity values were converted to optical density units. Next, targeted principal component analysis was used to detect and remove motion artifacts (tMotion = 1, tMask = 1, STDEVthresh = 50, AMPthresh = 0.5, nSV = 0.97, maxIter = 5). The data were then scanned again for remaining motion artifacts (tMotion = 1, tMask = 1, STDEVthresh = 50, AMPthresh = 0.5). The StimRejection function was used to reject uncorrected motion artifacts (tRange = −1 to 18). Finally, data were band‐pass filtered (hpf = 0.016, lpf = 0.5). Pre‐processed channel‐based optical density time‐series data were then used for the image reconstruction pipeline described below. Following pre‐processing, the percentage of periods lost was 0.50% for joint attention for caregivers, and 5.47% for joint attention and 5.36% for continued attention for infants. A total of 70 caregivers contributed fNIRS data for joint attention, 67 infants contributed fNIRS data for joint attention, and 62 infants contributed fNIRS data for continued attention.

##### 2.2.3.2 Converting Channel‐Based Data to Voxel‐Based Data

Channel‐based fNIRS data was converted to voxel‐wise fNIRS data using a recently‐validated image reconstruction pipeline (Forbes et al. 2021; Davidson et al. 2024; Amaireh et al. 2024; Theyer et al. 2024; Wijeakumar et al. 2023; Davidson, Caes, et al. 2023; Davidson, Shing, et al. 2023; Defenderfer et al. 2021). Image reconstruction is a useful approach for both sparse (as in the current study) and dense probe geometries because it considers information about the position of probe geometry, structural information where the probe sits on head models, how light would have traveled in those regions, and fNIRS data from the channels overlying these regions to generate voxel‐wise estimates of activation—information that is not considered in conventional channel‐based analyses. More generally, outcomes from image reconstruction approaches can be spatially aligned with fMRI data, aligned across age‐dependent cross‐sectional, longitudinal and lifespan datasets, and corrected for variability in probe positions, head sizes, and head shapes.

The steps of this pipeline are described below. First, to account for age‐specific differences in head size and head shape, age‐specific atlases (infants: at 6, 7.5, and 9 months; caregivers: at 20–24 , 25–29, 30–34 , 35–39, 40–44 , and 45–49 years) were identified and obtained from the Neurodevelopmental MRI database (Richards et al. 2016). Next, each atlas was segmented into a head model with four tissue types (scalp, cerebrospinal fluid, gray matter, and white matter). Then, scalp landmarks and positions of sources and detectors were digitized on a single adult wearing a 58 cm cap and a single child wearing a 48 cm cap using a Polhemus Patriot Digitizer. These digitized points were projected onto the surface of each age‐specific head model using AtlasViewerGUI in Homer2. Monte Carlo simulations with 1 million photons were run for each channel and for each infant and caregiver to estimate how fNIRS light would have traveled through each head model (called sensitivity profiles). These sensitivity profiles were inverted and integrated with pre‐processed channel‐based fNIRS data (described in Section 2.2.3.1) to generate voxel‐wise time‐series oxyhemoglobin (HbO) and deoxyhemoglobin (HbR) concentration data.

##### 2.2.3.3 Group‐Level Analyses

We first ran separate general linear models on the voxel‐wise time‐series data to generate a beta coefficient map for HbO concentration for joint attention for each caregiver, HbR concentration for joint attention for each caregiver, HbO concentration for joint attention for each infant, HbR concentration for joint attention for each infant, HbO concentration for continued attention for each infant, and HbR concentration for continued attention for each infant. For each model, each period was modeled with a 10 s boxcar using a hemodynamic response function (Eggebrecht et al. 2014). Resulting beta coefficient maps were registered to the MNI space and used in group analyses described below.

Only voxels that contained data from 65% of caregivers and infants were used in group analyses to generate reliable results. To extract significant regions of brain activation, voxel‐wise *t*‐tests comparing HbO and HbR concentration were run separately for joint attention in caregivers, joint attention in infants and continued attention in infants (using 3dttest++ in AFNI). The difference between HbO and HbR concentration was used because a difference driven by an increase in HbO concentration and a decrease in HbR concentration indicates “activation”, and a difference driven by a decrease in HbO concentration and an increase in HbR concentration indicates “suppression”. Activation in a brain region implies that the region was engaged in responding to or processing a stimulus/condition, and suppression implies that the region showed a reduced response or was inhibited in response to a specific stimulus/condition (Gazzaley et al. 2005; Suzuki and Gottlieb 2013). For example, if a region showed greater HbO concentration compared to HbR concentration for joint attention in caregivers, we can say that, that region was activated in caregivers during joint attention. The resultant *t*‐test images were thresholded at a cluster‐wise threshold of 10 voxels and a voxel‐wise *p* value of < 0.05 (using 3dClustSim in AFNI) to identify localized, significant brain regions. Once significant regions of brain activation were identified for joint attention in caregivers, joint attention in infants and continued attention in infants, average HbO and HbR concentration was extracted from voxels in these regions (using 3dROIstats in AFNI) and used in statistical analyses described in Section 2.4 to meet proposed objectives.

### 2.3 Infant Visual Short‐Term Memory

#### 2.3.1 Data Collection

To measure visual short‐term memory, infants were presented with an adapted version of the preferential looking short‐term memory task (Delgado Reyes et al. 2020; Ross‐Sheehy et al. 2003)—see Figure 1e. This task was presented on a 121 cm × 68 cm TV screen and ran on a laptop using PsychoPy software (v2021.2.3). Infants completed the task either seated in a high‐chair or on their caregiver's lap, approximately 120 cm away from the TV screen, in a dimly lit room. The caregiver was asked not to interact with their infant during the task. In this task, at the beginning of each trial, an attentional cue with an audible tone was presented at the center of the screen to engage the infant. Once the infant was engaged with the cue, the experimenter commenced the experimental trial. Each trial consisted of two side‐by‐side flashing displays of colored shapes (Drucker and Aguirre 2009; Wijeakumar et al. 2015). Between each flash, one shape randomly changed in color on one side (changing side) and on the other side, the colored shapes remained constant (non‐changing side) throughout the trial. The colors of the shapes differed from each other within each display. These colors were selected from a set of 16 colors (RGB values: 0 0 0; 255 255 255; 255 0 0; 0 255 0; 0 0 255; 255 255 0; 0 255 255; 255 0 255; 128 0 0; 128 128 0; 0 128 0; 128 0 128; 0 128 128; 0 0 128; 255 165 0; 222 184 135). The displays appeared on the screen for 500 ms and disappeared for 250 ms, repeatedly for a total of 10.25 s. There was an inter‐trial interval of 5 s between each trial. Memory load, that is, the number of shapes within each display was manipulated (1 shape—low load, 2 shapes—medium load or 3 shapes—high load).

If the infant stopped engaging with the task, a cartoon was displayed during the attentional cue to regain their attention. In most cases, this task was presented first, followed by the dyadic interactions. However, we worked around caregivers' preference and infant fussiness/fatigue. Families were always compensated for their time. Infants completed between one and six runs of the task, depending on their level of engagement and state of completion (mean: 3.17 ± 1.29).

This task was chosen because infants typically actively explore both displays and gradually develop a preference for the changing side and suppress the non‐changing side if the number of shapes presented on each display is within their short‐term memory capacity. By manipulating load (1, 2, or 3 items), we can assess memory capacity limits in infants. Temporal features of this task are also intended to reduce contributions from other memory processes (Ross‐Sheehy et al. 2003). The displays are presented briefly for 500 ms, which requires rapid memory formation and the delay period of 250 ms is long enough to prevent reliance on iconic memory but be within expected duration for short‐term memory processing. Additionally, the trial duration was set to 10 s to minimize contributions from long‐term memory, in line with more recent work using this paradigm (Davidson et al. 2024; Amaireh et al. 2024; Theyer et al. 2024; Delgado Reyes et al. 2020; Perone et al. 2011; Wijeakumar et al. 2019).

During the task, one video camera recorded the TV screen, and the other recorded the infants' face. These video recordings were then used to code looking behaviors. During the task, real‐time recordings were presented to the experimenter using Bandicam (https://www.bandicam.com/) and Movavi programs (https://www.movavi.com/mac/). DataVyu software was used to code looking behaviors from video recordings.

#### 2.3.2 Data Analyses

Video‐recordings were used to code infant eye‐movements. Each trial was first coded for the load and changing side. Following this, infant looks were coded as either “left” or “right”. Three coders were rigorously trained to accurately code trials and looks. Reliability was computed using Cohen's Kappa, a statistic that looks at percent agreement across categories normed by the base rate of each category. Twenty‐three percent of infants and caregivers were coded for reliability. Kappa values from 0.81 to 0.99 indicate near perfect agreement. The mean Kappa value across coders was 0.94.

From the coded looks, a change preference (CP) score was calculated as the amount of time spent looking toward the changing display, divided by the total looking duration. Higher CP scores indicated greater ability to detect and remain fixated on the changing display, and better visual short‐term memory. We used CP scores at the low load for further analyses as it has been shown to be linked to caregiver CP scores at the low load (Theyer et al. 2024) and was therefore relevant for caregiver‐infant dyadic interactions.

### 2.4 Statistical Analyses

Summary statistics for dyadic behavior and brain analyses, and behavioral analyses for the infant PLT are reported in Supporting Information S1: Tables S1 and S2, respectively. RStudio (v2023.03.0) was used for all statistical analyses. Linear models were used to examine associations for meeting all objectives that is between duration of joint attention and continued attention, between brain function in caregivers and brain function in infants, between brain function measures and duration of attention periods, and between duration and brain function during attention periods, and infant visual short‐term memory. The specific models are described in Section 3 below. Infant age in days was used as a covariate in all models.

## 3 Results

**FIGURE 3:**
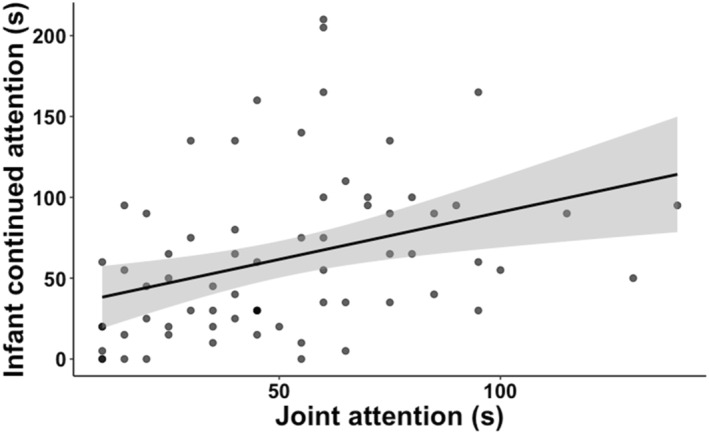
Association between joint attention in caregivers and infants and continued attention in infants.

The first objective was to examine whether duration of joint attention was linked to duration of continued attention in infants. To address this, a linear effects model with duration of joint attention as predictor and duration of continued attention as outcome was run. There was a significant positive association that is longer duration of joint attention in caregivers and infants was associated with longer duration of continued attention in infants (*F*_(2,66)_ = 5.08, *p* = 0.003, *η* ^2^ = 0.13)—see Figure 3.

**FIGURE 4:**
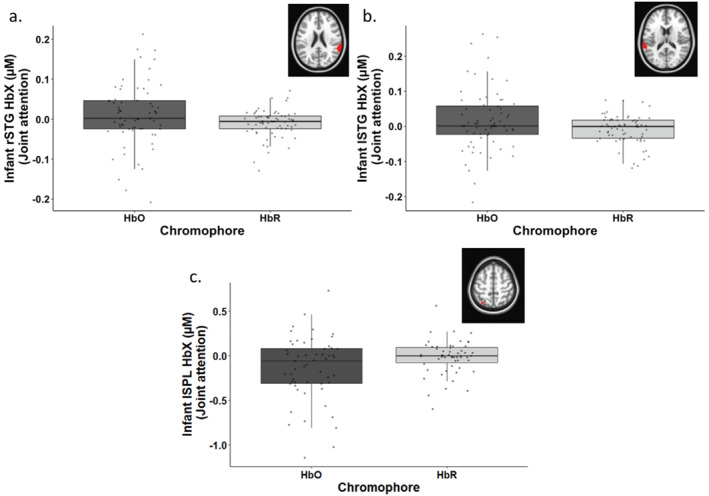
Activation in (a) rSTG and (b) lSTG and suppression in (c) lSPL during joint attention in infants.

The second objective was to identify key brain regions engaged during joint attention in caregivers and infants and continued attention in infants. To do this, voxel‐wise *t*‐tests comparing HbO and HbR concentration were run separately for joint attention in caregivers, joint attention in infants and continued attention in infants (details presented in Section 2.2.3.3). Significant clusters were only observed for joint attention in infants. Specifically, there was a difference between HbO and HbR concentration in the right superior temporal gyrus (rSTG), left superior temporal gyrus (lSTG) and left superior parietal lobule (lSPL)—see Table 3. In the lSTG and rSTG, HbO concentration was significantly greater than HbR concentration (rSTG: *p* = 0.048; lSTG: *p* = 0.030)—see Figure 4a,b. In the lSPL, HbR concentration was significantly greater than HbO concentration (*p* = 0.025)—see Figure 4c. There were no significant clusters for joint attention in caregivers and continued attention in infants. For meeting the third, fourth, and fifth objectives of the study, we extracted HbO concentration from these regions for joint attention in caregivers and continued attention in infants (as well as joint attention in infants). Exploratory analyses run on HbR concentration from these regions for the third, fourth and fifth objectives are reported in the Supporting Information S1.

**TABLE 3:** Significant clusters showing a difference between HbO and HbR concentration in joint attention in infants.

Model	Effect	Cluster	Size (mm^3^)	MNI coordinates		
				Center of mass		
				*x*	*y*	*z*
Joint attention in infants	Difference between HbO and HbR concentration	Right superior temporal gyrus (rSTG)	381	−62.4	+38.5	+21.3
		Left superior temporal gyrus (lSTG)	212	+62.1	+36.0	+15.3
		Left superior parietal lobule (lSPL)	117	+27.1	+65.2	+58.1

**FIGURE 5:**
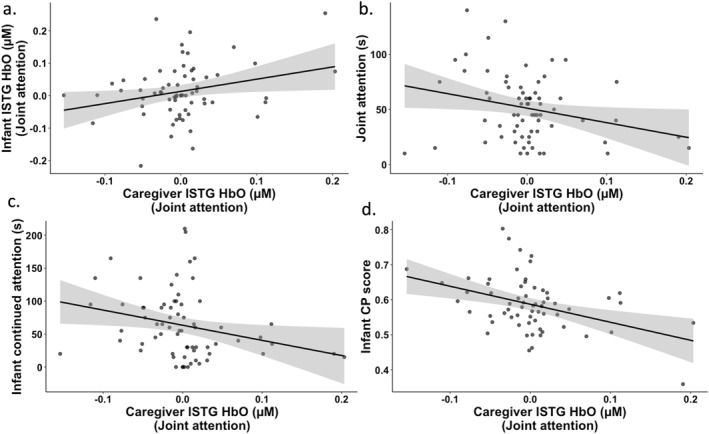
Reduced lSTG HbO concentration in caregivers during joint attention was associated with (a) reduced lSTG HbO concentration in infants during joint attention, (b) longer duration of joint attention, (c) longer duration of continued attention in infants, and (d) higher CP scores in infants.

The third objective was to examine whether there was an association between brain function in caregivers and brain function in infants during joint attention, and whether this brain function was related to duration of joint attention. We ran three linear effects models each for lSTG HbO concentration, rSTG HbO concentration and lSPL HbO concentration. In the first model, we examined whether HbO concentration in caregivers during joint attention was associated with HbO concentration in infants during joint attention. In the second model, we examined whether HbO concentration in caregivers was associated with duration of joint attention. In the third model, we examined whether HbO concentration in infants during joint attention was associated with duration of joint attention. We found two significant associations only for lSTG. Specifically, reduced lSTG HbO concentration in caregivers during joint attention was associated with reduced lSTG HbO concentration in infants during joint attention (*F*_(2,63)_ = 3.54, *p* = 0.037, *η* ^2^ = 0.07)—see Figure 5a. Further, reduced lSTG HbO concentration in caregivers during joint attention was associated with longer duration of joint attention (*F*_(2,65)_ = 2.65, *p* = 0.038, *η* ^2^ = 0.06)—see Figure 5b.

The fourth objective was to examine whether duration of continued attention in infants was associated with brain function in infants during continued attention, brain function in caregivers during joint attention and brain function in infants during joint attention. We ran three linear effects models each for lSTG HbO concentration, rSTG HbO concentration and lSPL HbO concentration. In the first model, we examined whether duration of continued attention was associated with HbO concentration in infants during continued attention. In the second model, we examined whether duration of continued attention was associated with HbO concentration in infants during joint attention. In the third model, we examined whether duration of continued attention was associated with HbO concentration in caregivers during joint attention. We found one significant association only for lSTG. Specifically, longer duration of continued attention in infants was associated with reduced lSTG HbO concentration in caregivers during joint attention (*F*_(2,66)_ = 3.03, *p* = 0.023, *η* ^2^ = 0.08)—see Figure 5c.

The fifth objective was to examine whether key behavioral and brain measures emerging from meeting afore‐mentioned objectives were associated with better visual short‐term memory in infants. Here, we focus on lSTG because there were caregiver‐infant, and brain‐behavior associations in this region—thus, we were wanted to examine whether this region was also related to infant visual short‐term memory. Thus, four key measures were used based on significant effects in objectives 1–4 that is for duration of joint attention, duration of continued attention, lSTG HbO concentration in caregivers during joint attention, and lSTG HbO concentration in infants during joint attention. Thus, four models were run. In the first model, we examined whether duration of joint attention was associated with infant CP score. In the second model, we examined whether duration of continued attention was associated infant CP score. In the third model, we examined whether lSTG HbO concentration in caregivers during joint attention was associated with infant CP score. In the fourth model, we examined whether lSTG HbO concentration in infants during joint attention was associated with infant CP score. Note that these measures were not included in the same model as most of them were related to each other (see previous sections). There was only one significant association. Specifically, reduced lSTG HbO concentration in caregivers during joint attention was associated with a higher infant CP scores (*F*_(2,61)_ = 6.12, *p* = 0.001, *η* ^2^ = 0.16)—see Figure 5d. Exploratory analyses were also run using HbO and HbR data from rSTG and lSPL clusters—these are reported in the Supporting Information S1.

## 4 Discussion

The over‐arching objective of the current study was to examine brain function in infants and caregivers during attention periods in dyadic interactions, and whether extent of engagement and brain function during these periods were associated with visual short‐term memory in infants. We discuss our findings below.

The first objective was to examine whether duration of joint attention was linked to duration of continued attention in infants. We found that longer duration of joint attention between caregivers and infants was associated with longer duration of continued attention in infants. These findings are in line with previous studies using head‐mounted eye‐tracking to observe free‐play interactions in caregivers and their toddlers. In one such study, the authors reported that sustained attention episodes that occurred alongside caregiver joint attention were significantly longer than those episodes that occurred without joint attention (Yu and Smith 2016). In another study observing caregiver‐infant interactions in homes, play bouts that included joint engagement between caregivers and infants were longer compared to bouts without any caregiver input (Schatz et al. 2022). These behavioral links set the precedence for exploring underlying brain function.

Our second objective was to identify brain regions engaged during joint attention in caregivers, joint attention in infants, and continued attention in infants. We observed engagement in bilateral STG and lSPL in infants during joint attention. The STG has been implicated in several aspects of social cognition and perception (Deen et al. 2015), including perception of voices (Belin et al. 2000) and motion (Bonda et al. 1996; Pelphrey et al. 2005; Grossman and Blake 2002; Allison et al. 2000), visual attention to salient events (Corbetta and Shulman 2002), theory of mind (Gallagher et al. 2000; Saxe and Powell 2006; Saxe and Kanwisher 2003; Ciaramidaro et al. 2007) and understanding actions of others, such as gaze shifts (Pelphrey et al. 2003) and reaching (Pelphrey et al. 2004). For example, one study used eye‐tracking and fMRI in children to examine brain activation while they either initiated or responded to gaze interactions with a partner presented on a screen. The authors reported joint attention‐related activation in a cluster which extended to the left temporal lobe (Oberwelland et al. 2016). Interestingly, temporo‐parietal patterns have been reported in experimental studies of attention and visual short‐term/working memory in adults and infants. For instance, one study found increasing parietal activation and temporal suppression with increasing working memory demands in adults (Todd and Marois 2004). In another study, infants demonstrated parietal activation and temporal suppression when engaging with a preferential looking visual short‐term memory task (Wijeakumar et al. 2023). Taken together, infants appear to engage brain regions involved in meeting attention and short‐term memory demands, even during joint attention episodes with a social partner.

While we observed significant effects in infants that align with findings in older children and adults, we did not find any significant clusters of activation during joint attention in caregivers and continued attention in infants. Absence of effects in adults does not align with previous work. For example, left temporal activation has been reported when adults jointly engage with gaze behaviors of a partner presented on a monitor in fMRI studies (Redcay et al. 2012; Schilbach et al. 2010). In the current study, it is possible that our fNIRS channels did not directly overlap with key regions in the temporal cortex involved in joint attention in adults or continued attention in infants. Nonetheless, activation extracted from recorded temporal regions such as the lSTG during joint attention in caregivers and continued attention in infants yielded significant effects suggesting that this area might still be important for dyadic interactions—these findings are discussed below.

Collective findings from the third and fourth objectives highlight the importance of caregiver brain function during dyadic interactions. The third objective was to examine whether there was an association between brain function in caregivers and brain function in infants during joint attention, and how these brain function measures were linked to duration of joint attention. The fourth objective was to examine whether duration of continued attention in infants was associated with brain function in infants during continued attention, brain function in infants during joint attention, and brain function in caregivers during joint attention. Our findings revealed that caregivers who showed reduced lSTG activation also had infants who showed reduced lSTG activation during joint attention. Spatio‐functional lSTG alignment in caregivers and their infants' might be explained by heritability. For example, findings from twin studies have demonstrated structural heritability in the temporal cortex in children (Lenroot et al. 2009; van der Meulen et al. 2020). The temporo‐parietal junction also demonstrates additional shared environmental influences, suggesting that this region is also sensitive to social experiences (van der Meulen et al. 2020). Along a similar vein, studies have reported pronounced neural synchrony in the temporal cortex between caregivers and children during cooperative interactions (Nguyen et al. 2020; Jiang et al. 2012; Miller et al. 2019).

We also found links between lSTG activation in caregivers and duration of joint attention and continued attention periods. Critically, caregivers with reduced lSTG activation during joint attention engaged in longer periods of joint attention with their infants and had infants who also demonstrated longer periods of continued attention. It is possible that in adults, this region might need to be suppressed to efficiently maintain and update items/goals/actions in short‐term/working memory and inhibit distractions and irrelevant stimuli. Further, suppression of the temporal cortex might be necessary for enhancing engagement in fronto‐parietal regions underlying these critical cognitive functions, which then impacts the duration of joint and continued attention. Interestingly, exploratory analyses revealed the opposite pattern between infant lSTG engagement and duration of continued attention—based on HbR concentration (refer to exploratory analyses in Supporting Information S1)—it is unclear whether this is reflective of developmental differences in socio‐cognitive abilities or physiology. Future work should conduct longitudinal and cross‐sectional investigations to examine this further.

Our final objective was to examine whether caregiver‐infant dyadic behaviors and brain function were linked to infant short‐term memory. While we found no links with duration of attentional episodes, infant short‐term memory performance was robustly linked to caregiver and infant brain mechanisms during joint attention in dyadic interactions. Specifically, better short‐term memory performance in infants was linked to reduced lSTG activation in caregivers and increased lSPL activation in infants during joint attention (refer to exploratory analyses in Supporting Information S1). More generally, experimental neuroimaging studies have shown suppression in the temporal cortex and activation in the parietal cortex linked to short‐term/working memory processing (Wijeakumar et al. 2023; Todd et al. 2005). In line with the interpretation of the findings in previous objectives, it is possible that caregivers who are better at suppressing lSTG during play interactions, can more promptly pick up on cues, and guide and shape their infants' cognitive abilities resulting in longer bouts of joint attention and continued attention—eventually, improving infants' visual short‐term memory. Interestingly, left‐lateralized parietal cortex has been directly implicated in previous work using similar experimental short‐term memory tasks in infants. For example, the left anterior intraparietal sulcus has been shown to be involved in short‐term memory processing in 6‐ and 9‐month‐old infants (Wijeakumar et al. 2023). In another study, lSPL activation in infants during short‐term memory processing was positively associated with short‐term memory processing in their caregivers (Theyer et al. 2024). Taken together, our findings suggest that brain mechanisms involved in dyadic interactions might be important for shaping infant cognitive function.

## 5 Strengths and Limitations

The current study offers some unique strengths. First, it is one of few studies that focussed on task‐based brain function in caregivers and infants during attention periods in dyadic interactions. Second, the study presents evidence tying caregiver brain function to infant brain function, and duration of attention engagement. Third, the study contributes to bridging the gap between caregiver‐child engagement during dyadic interactions and infant visual short‐term memory.

There are some limitations in the current study that must be considered and addressed in future studies. First, future studies should consider increasing fNIRS channels to cover more temporal areas and investigate whether this extended coverage reveals significant regions of activation during joint attention in caregivers and continued attention in infants. Second, we were unable to digitize scalp landmarks and locations of sources and detectors on every dyad to account for differences in head size, head shapes and differences in position of probe geometry across participants. Future work should employ more efficient methods to account for such differences. Third, our interactions lasted for a maximum of 7 min. Future work should aim to collect interaction data for a longer duration to gather more events for each attention period.

## 6 Conclusions

The over‐arching objective of the current study was to examine brain function in infants and caregivers during critical attention periods in dyadic interactions, and whether extent of engagement and brain function during these periods were associated with visual short‐term memory in infants. In line with previous findings, duration of joint attention between caregivers and infants was linked to duration of continued attention in infants. Importantly, reduced lSTG activation in caregivers during joint attention was associated with reduced lSTG activation in infants during joint activation, longer duration of joint and continued attention, and better visual short‐term memory in infants. In addition, exploratory analyses revealed that greater infant lSPL engagement during joint attention was also associated with better visual short‐term memory performance. Our findings contribute to the understanding of cortical mechanisms engaged during attention periods in caregiver‐infant interactions, and importantly, how these mechanisms might be linked to visual short‐term memory in infants.

## Author Contributions

**Aimee Theyer:** data curation, formal analysis, methodology, visualization, writing – original draft, writing – review and editing. **Sobanawartiny Wijeakumar:** conceptualization, data curation, formal analysis, funding acquisition, investigation, methodology, project administration, software, supervision, visualization, writing – original draft, writing – review and editing.

## Conflicts of Interest

The authors declare no conflicts of interest.

## Supporting information


Supporting Information S1

## Data Availability

The data that support the findings of this study are available on request from the corresponding author.
