# Supplementary material for: Brain‐Behavior Associations During Interactions Between Caregivers and Infants
Source: Infancy. 2025 Sep 3;30(5):e70044. doi: 10.1111/infa.70044 (PMC12408883; doi:10.1111/infa.70044)
Supplement: Supplementary file 1 — Supporting Information S1 [file INFA-30-0-s001.docx]

**Supplementary materials**

**Supplementary Table 1.** Summary statistics for dyadic behaviour and brain analyses. Note that window length was fixed at 10 seconds for fNIRS analyses, thus duration, SD, and range information are not relevant for dyadic brain. Also note that while some periods of infant continued attention were 0, these events were not included in brain analyses; only periods that provided at least 5 seconds of data were included for brain analyses.

| **Analyses** | **Category** | **No. of dyads/subjects** | **Mean infant age (days)** | **Mean no. of periods per dyad** | **Mean duration per dyad (s)** | **SD (seconds)** | **Range (seconds)** |
| --- | --- | --- | --- | --- | --- | --- | --- |
| **Dyadic behaviour** | **Caregiver-infant joint attention** | 69 | 251 | 2.81 | 50.94 | 30.00 | 10 - 140 |
|  | **Infant continued attention** | 70 | 250 | 2.86 | 63.07 | 50.18 | 0 - 210 |
| **Dyadic brain** | **Caregiver joint attention** | 70 | 251 | 2.86 |  |  |  |
|  | **Infant joint attention** | 67 | 251 | 2.84 |  |  |  |
|  | **Infant continued attention** | 62 | 251 | 2.57 |  |  |  |

**Supplementary Table 2.** Summary statistics for behavioural analyses for the infant PLT.

| **Category** | **No. of infants** | **Mean infant age (days)** | **Mean no. of trials** | **Mean score** | **SD** | **Range** |
| --- | --- | --- | --- | --- | --- | --- |
| **CP score (low load)** | 86 | 251 | 12.59 | 0.59 | 0.08 | 0.36 - 0.80 |

**Statistical models used in analyses.**

**Objective 1:** To address the first objective, we ran a linear model with duration of joint attention (JA) as the predictor, duration of continued attention (CA) as the outcome, and Infant age (I_Age) as the covariate:

model1 <- lm(CA ~ JA + I_Age, data = test)

car::Anova(model1, type = 'III')

summary(model1)

effectsize::eta_squared(car::Anova(model1, type = 'III'), alternative = 'two.sided')

**Objective 2:** To address the second objective, we performed a paired voxel-wise t-test in AFNI (*3dttest++*) comparing HbO and HbR. This was done separately for infant joint attention, caregiver joint attention, and infant sustained attention. The following example is for infant joint attention (I_JA). The script included the HbO and HbR files for each participant (e.g., I1_JA, I2_JA etc.):

3dttest++ \

-prefix ttest_I_JA \

-mask GroupMask.nii \

-labelA J_HbO \

-setA \

I1_JA_Unmasked_HbO_ND_To_Atlas_ClipToBrain.nii.gz \

I2_JA_Unmasked_HbO_ND_To_Atlas_ClipToBrain.nii.gz \

…

-labelB J_HbR \

-setB \

I1_JA_Unmasked_HbR_ND_To_Atlas_ClipToBrain.nii.gz \

I2_JA_Unmasked_HbR_ND_To_Atlas_ClipToBrain.nii.gz \

…

-paired \

-Clustsim \

-ETAC \

-prefix_clustsim cc

**Objective 3:** To address the third objective, we ran three linear models each for lSTG HbO concentration, rSTG HbO concentration, and lSPL HbO concentration. In the first model, HbO concentration in caregivers during joint attention (HbO_C_JA) was the predictor, HbO concentration in infants during joint attention (HbO_I_JA) was the outcome, and infant age (I_Age) was the covariate:

model1 <- lm(HbO_I_JA ~ HbO_C_JA + I_Age, data = test)

car::Anova(model1, type = 'III')

summary(model1)

effectsize::eta_squared(car::Anova(model1, type = 'III'), alternative = 'two.sided')

In the second model, HbO concentration in caregivers during joint attention (HbO_C_JA) was the predictor, duration of joint attention (JA) was the outcome, and infant age (I_Age) was the covariate:

model1 <- lm(JA ~ HbO_C_JA + I_Age, data = test)

car::Anova(model1, type = 'III')

summary(model1)

effectsize::eta_squared(car::Anova(model1, type = 'III'), alternative = 'two.sided')

In the third model, HbO concentration in infants during joint attention (HbO_I_JA) was the predictor, duration of joint attention (JA) was the outcome, and infant age (I_Age) was the covariate:

model1 <- lm(JA ~ HbO_I_JA + I_Age, data = test)

car::Anova(model1, type = 'III')

summary(model1)

effectsize::eta_squared(car::Anova(model1, type = 'III'), alternative = 'two.sided')

**Objective 4:** To address the fourth objective, we ran three linear models each for lSTG HbO concentration, rSTG HbO concentration, and lSPL HbO concentration. In the first model, HbO concentration in infants during continued attention (HbO_I_CA) was the predictor, duration of continued attention (CA) was the outcome, and infant age (I_Age) was the covariate:

model1 <- lm(CA ~ HbO_I_CA + I_Age, data = test)

car::Anova(model1, type = 'III')

summary(model1)

effectsize::eta_squared(car::Anova(model1, type = 'III'), alternative = 'two.sided')

In the second model, HbO concentration in infants during joint attention (HbO_I_JA) was the predictor, duration of continued attention (CA) was the outcome, and infant age (I_Age) was the covariate:

model1 <- lm(CA ~ HbO_I_JA + I_Age, data = test)

car::Anova(model1, type = 'III')

summary(model1)

effectsize::eta_squared(car::Anova(model1, type = 'III'), alternative = 'two.sided')

In the third model, HbO concentration in caregivers during joint attention (HbO_C_JA) was the predictor, duration of continued attention (CA) was the outcome, and infant age (I_Age) was the covariate:

model1 <- lm(CA ~ HbO_C_JA + I_Age, data = test)

car::Anova(model1, type = 'III')

summary(model1)

effectsize::eta_squared(car::Anova(model1, type = 'III'), alternative = 'two.sided')

**Objective 5:** To address the fifth objective, we ran four linear models. In the first model, duration of joint attention (JA) was the predictor, infant CP score (CP) was the outcome, and infant age (I_Age) was the covariate:

model1 <- lm(CP ~ JA + I_Age, data = test)

car::Anova(model1, type = 'III')

summary(model1)

effectsize::eta_squared(car::Anova(model1, type = 'III'), alternative = 'two.sided')

In the second model, duration of continued attention (CA) was the predictor, infant CP score (CP) was the outcome, and infant age (I_Age) was the covariate:

model1 <- lm(CP ~ CA + I_Age, data = test)

car::Anova(model1, type = 'III')

summary(model1)

effectsize::eta_squared(car::Anova(model1, type = 'III'), alternative = 'two.sided')

In the third model, lSTG HbO concentration in caregivers during joint attention (lSTG_HbO_C_JA) was the predictor, infant CP score (CP) was the outcome, and infant age (I_age) was the covariate:

model1 <- lm(CP ~ lSTG_HbO_C_JA + I_Age, data = test)

car::Anova(model1, type = 'III')

summary(model1)

effectsize::eta_squared(car::Anova(model1, type = 'III'), alternative = 'two.sided')

In the fourth model, lSTG HbO concentration in infants during joint attention (lSTG_HbO_I_JA) was the predictor, infant CP score (CP) was the outcome, and infant age (I_age) was the covariate:

model1 <- lm(CP ~ lSTG_HbO_I_JA + I_Age, data = test)

car::Anova(model1, type = 'III')

summary(model1)

effectsize::eta_squared(car::Anova(model1, type = 'III'), alternative = 'two.sided')

**Statistical models used in exploratory analyses.**

**Objective 3:** In relation to the third objective, we also ran three exploratory linear models each for lSTG HbR concentration, rSTG HbR concentration, and lSPL HbR concentration. In the first model, HbR concentration in caregivers during joint attention (HbR_C_JA) was the predictor, HbR concentration in infants during joint attention (HbR_I_JA) was the outcome, and infant age (I_Age) was the covariate:

model1 <- lm(HbR_I_JA ~ HbR_C_JA + I_Age, data = test)

car::Anova(model1, type = 'III')

summary(model1)

effectsize::eta_squared(car::Anova(model1, type = 'III'), alternative = 'two.sided')

In the second model, HbR concentration in caregivers during joint attention (HbR_C_JA) was the predictor, duration of joint attention (JA) was the outcome, and infant age (I_Age) was the covariate:

model1 <- lm(JA ~ HbR_C_JA + I_Age, data = test)

car::Anova(model1, type = 'III')

summary(model1)

effectsize::eta_squared(car::Anova(model1, type = 'III'), alternative = 'two.sided')

In the third model, HbR concentration in infants during joint attention (HbR_I_JA) was the predictor, duration of joint attention (JA) was the outcome, and infant age (I_Age) was the covariate:

model1 <- lm(JA ~ HbR_I_JA + I_Age, data = test)

car::Anova(model1, type = 'III')

summary(model1)

effectsize::eta_squared(car::Anova(model1, type = 'III'), alternative = 'two.sided')

**Objective 4:** In relation to the fourth objective, we also ran three exploratory linear models each for lSTG HbR concentration, rSTG HbR concentration, and lSPL HbR concentration. In the first model, HbR concentration in infants during continued attention (HbR_I_CA) was the predictor, duration of continued attention (CA) was the outcome, and infant age (I_Age) was the covariate:

model1 <- lm(CA ~ HbR_I_CA + I_Age, data = test)

car::Anova(model1, type = 'III')

summary(model1)

effectsize::eta_squared(car::Anova(model1, type = 'III'), alternative = 'two.sided')

In the second model, HbR concentration in infants during joint attention (HbR_I_JA) was the predictor, duration of continued attention (CA) was the outcome, and infant age (I_Age) was the covariate:

model1 <- lm(CA ~ HbR_I_JA + I_Age, data = test)

car::Anova(model1, type = 'III')

summary(model1)

effectsize::eta_squared(car::Anova(model1, type = 'III'), alternative = 'two.sided')

In the third model, HbR concentration in caregivers during joint attention (HbR_C_JA) was the predictor, duration of continued attention (CA) was the outcome, and infant age (I_Age) was the covariate:

model1 <- lm(CA ~ HbR_C_JA + I_Age, data = test)

car::Anova(model1, type = 'III')

summary(model1)

effectsize::eta_squared(car::Anova(model1, type = 'III'), alternative = 'two.sided')

**Objective 5:** In relation to the fifth objective, we also ran a further 16 exploratory linear models to check effects on HbO data in rSTG and lSPL, and HbR data from lSTG, rSTG, and lSPL. First, five individual models were run, each examining caregiver lSPL HbO, rSTG HbO, lSPL HbR, rSTG HbR, and lSTG HbR concentration during joint attention. In these models, caregiver HbO/HbR concentration during joint attention (HbX_C_JA) was the predictor, infant CP score (CP) was the outcome, and infant age (I_Age) was the covariate.

model1 <- lm(CP ~ HbX_C_JA + I_Age, data = test)

car::Anova(model1, type = 'III')

summary(model1)

effectsize::eta_squared(car::Anova(model1, type = 'III'), alternative = 'two.sided')

Next, five individual models were run, each examining infant lSPL HbO, rSTG HbO, lSPL HbR, rSTG HbR, and lSTG HbR concentration during joint attention. In these models, infant HbO/HbR concentration during joint attention (HbX_I_JA) was the predictor, infant CP score (CP) was the outcome, and infant age (I_age) was the covariate.

model1 <- lm(CP ~ HbX_I_JA + I_Age, data = test)

car::Anova(model1, type = 'III')

summary(model1)

effectsize::eta_squared(car::Anova(model1, type = 'III'), alternative = 'two.sided')

Finally, six individual models were run, each examining infant lSPL HbO, rSTG HbO, lSTG HbO, lSPL HbR, rSTG HbR, and lSTG HbR concentration during continued attention. In these models, infant HbO/HbR concentration during continued attention (HbX_I_CA) was the predictor, infant CP score (CP) was the outcome, and infant age (I_age) was the covariate.

model1 <- lm(CP ~ HbX_I_CA + I_Age, data = test)

car::Anova(model1, type = 'III')

summary(model1)

effectsize::eta_squared(car::Anova(model1, type = 'III'), alternative = 'two.sided')

**Exploratory analyses.** Here, we present findings using HbR data from all clusters for the third, fourth and fifth objectives, and exploratory analyses using HbO and HbR data from rSTG and lSPL clusters for the fifth objective.

The third objective was to examine whether there was an association between brain function in caregivers and brain function in infants during joint attention, and whether this brain function was related to duration of joint attention. As part of exploratory analyses on HbR concentration data, we ran three linear models each for lSTG HbR concentration, rSTG HbR concentration and lSPL HbR concentration. In the first model, we examined whether HbR concentration in caregivers during joint attention was associated with HbR concentration in infants during joint attention. In the second model, we examined whether HbR concentration in caregivers during joint attention was associated with duration of joint attention. In the third model, we examined whether HbR concentration in infants during joint attention was associated with duration of joint attention. We found two significant associations. Specifically, reduced lSPL HbR concentration in caregivers during joint attention was associated with increased lSPL HbR concentration in infants during joint attention (*F*_(2,12)_ = 2.94, *p* = .033, η^2^=.32) – see Supplementary Figure 1a. Note that for the lSPL cluster, spatial overlap was not observed for all caregiver-infant dyads, resulting in a smaller number of data-points. Second, reduced lSTG HbR concentration in infants during joint attention was associated with longer duration of joint attention (*F*_(2,62)_ = 2.94, *p* = .038, η^2^=.07) – see Supplementary Figure 1b.


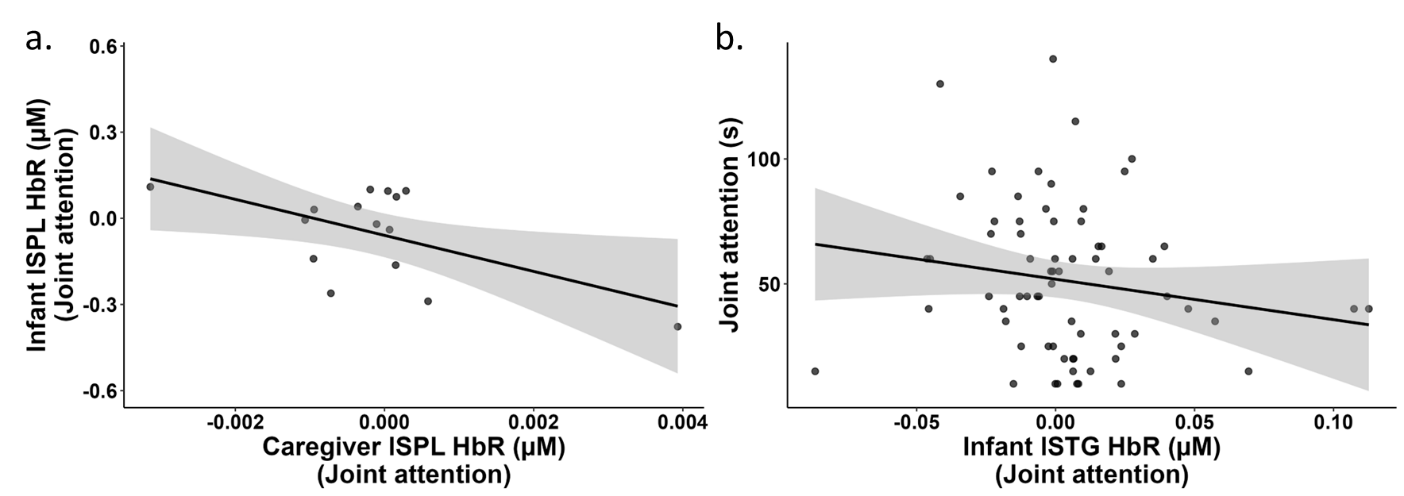


**Supplementary Figure 1.** (a) Increased lSPL HbR concentration in caregivers during joint attention was associated with decreased lSPL HbR concentration in infants during joint attention and (b) reduced lSTG HbR concentration in infants during joint attention was associated with longer duration of joint attention. Note that for the lSPL cluster, spatial overlap was not observed for all caregiver-infant dyads, resulting in a smaller number of data-points.

The fourth objective was to examine whether duration of continued attention in infants was associated with brain function in infants during continued attention, brain function in caregivers during joint attention and brain function in infants during joint attention. As part of exploratory analyses on HbR concentration data, we ran three linear models each for lSTG HbR concentration, rSTG HbR concentration and lSPL HbR concentration. In the first model, we examined whether duration of continued attention was associated with HbR concentration in infants during continued attention. In the second model, we examined whether duration of continued attention was associated with HbR concentration in infants during joint attention. In the third model, we examined whether duration of continued attention was associated with HbR concentration in caregivers during joint attention. We found two significant associations. Specifically, longer duration of continued attention in infants was associated with increased lSTG HbR concentration in infants during joint attention (*F*_(2,63)_ = 3.98, *p* = .010, η^2^=.10) – see Supplementary Figure 2a. Further, longer duration of continued attention in infants was associated with decreased lSTG HbR concentration in caregivers during joint attention (*F*_(2,66)_ = 2.38, *p* = .047, η^2^=.06) – see Supplementary Figure 2b.


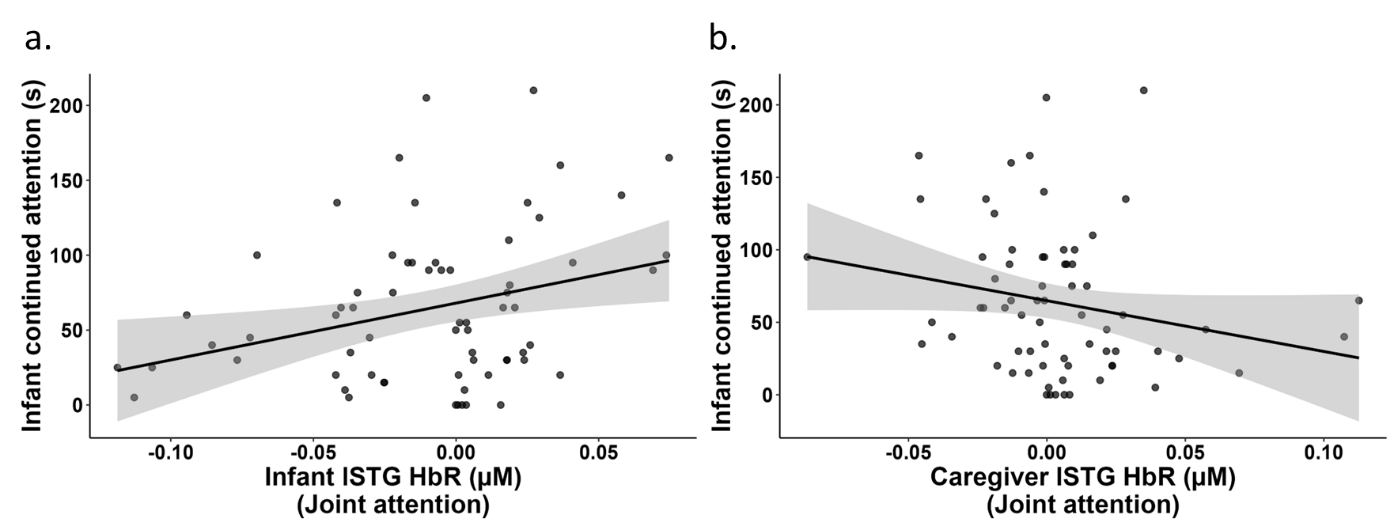


**Supplementary Figure 2.** (a) Increased lSTG HbR concentration in infants during joint attention was associated with longer duration of infant continued attention and (b) reduced lSTG HbR concentration in caregivers was associated with longer duration of infant continued attention.

The fifth objective was to examine whether key behavioural and brain measures emerging from meeting afore-mentioned objectives were associated with better visual cognition in infants. Considering significant effects from afore-mentioned exploratory analyses, a total of 16 models were run. First, we examined whether brain function in caregivers during joint attention was associated with infant CP score. Here, 5 individual models were run each for lSPL HbO, rSTG HbO, lSPL HbR, rSTG HbR, and lSTG HbR. Next, we examined whether brain function in infants during joint attention was associated with infant CP score. Here, 5 individual models were run each for lSPL HbO, rSTG HbO, lSPL HbR, rSTG HbR, and lSTG HbR. Finally, we examined whether brain function in infants during continued attention was associated with infant CP score. Here, 6 individual models were run each for lSPL HbO, rSTG HbO, lSTG HbO, lSPL HbR, rSTG HbR, and lSTG HbR. There was only one significant association. Specifically, increased lSPL HbO concentration in infants during joint attention was associated with higher infant CP scores (*F*_(2,47)_ = 2.60, *p* = .035, η^2^=.09) – see Supplementary Figure 3.


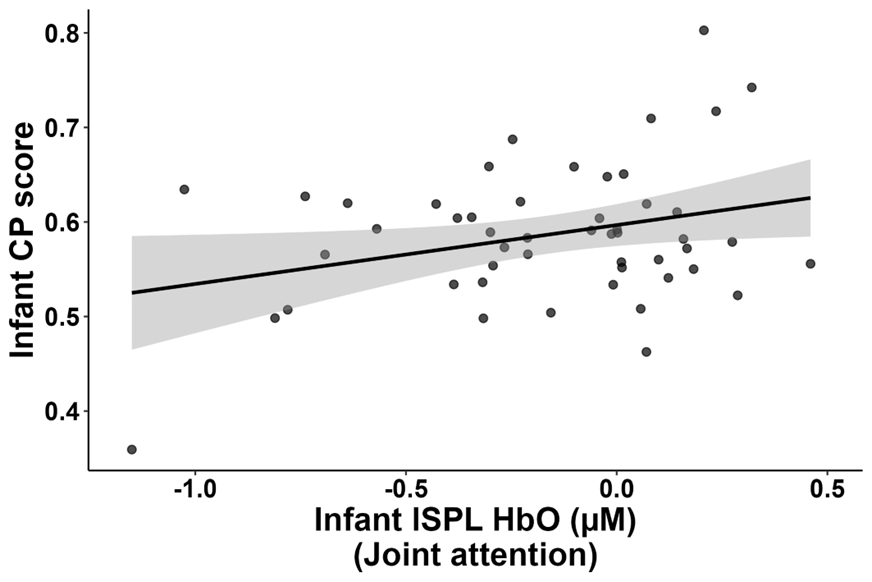


**Supplementary Figure 3.** Increased lSPL HbO concentration in infants during joint attention was associated with higher CP scores in infants.
